# The Mediating Effect of Alexithymia on the Relationship Between Schizotypal Traits and Sleep Problems Among College Students

**DOI:** 10.3389/fpsyt.2020.00153

**Published:** 2020-03-04

**Authors:** Qianwen Ma, Xiaoxiang Zhang, Laiquan Zou

**Affiliations:** Chemical Senses and Mental Health Lab, Department of Psychology, School of Public Health, Southern Medical University, Guangdong Provincial Key Laboratory of Tropical Disease Research, Guangzhou, China

**Keywords:** alexithymia, schizotypal traits, insomnia, sleep problems, college students, mediating effect

## Abstract

A body of research has investigated the relationship between alexithymia and sleep problems, as well as the relationship between schizophrenia and alexithymia. However, there have been few studies on the relationships between the three. The current study explored the relationship between schizotypal traits and sleep problems among college students, and the potential role of alexithymia as a mediator of this relationship. The participants were all first-year students at a medical university in Guangdong province, China. A total of 2,626 college students participated. They were asked to complete a questionnaire that incorporated the Schizotypal Personality Questionnaire (SPQ), the Toronto Alexithymia Scale (TAS-20), and the Insomnia Severity Index (ISI). The results revealed a relatively high percentage of students with mild insomnia (74.8%) and a smaller percentage with moderate to severe insomnia (7.9%). Correlation analysis revealed that both the TAS-20 and ISI scores had significant positive correlations with the SPQ score (*p* < 0.01). There was also a significant positive correlation between the TAS-20 and ISI scores (*p* < 0.01). The ISI score was significantly influenced by the SPQ score in a direct way, and increased considerably with increases in the TAS-20 score, indicating the importance of alexithymia as a mediator. The mediation model was tested via regression analysis and the bias-corrected bootstrap method, and these results further confirmed the role of alexithymia as a mediator.

## Introduction

Sleep problems can be found in 30–80% of schizophrenia patients (1). They are defined as increased sleep latency, frequent night waking, shorter sleep duration, and other parasomnias (2). Additionally, studies have found that schizotypal traits are present in people without schizophrenia, including offspring with parental psychiatric disorders and the general population (3). Schizotypal traits are of interest and importance in their own right but also have theoretical and clinical relevance to schizophrenia (4). Individuals with schizotypal traits show similar symptoms to schizophrenia patients but to a milder degree. These traits include social withdrawal, reduced cognitive capacity, and affective dysregulation. Most researchers in psychopathology hold the view that schizotypy is a construct that is intimately connected to a schizophrenia-related liability (5). Evidence also suggests that individuals with high levels of schizotypal traits resemble schizophrenia patients in experiencing much more vivid nightmares or pleasant dreams than other individuals (6). Moreover, sleep problems have been associated with psychosis-like experiences in epidemiological studies (7). A growing amount of evidence suggests that recognizing and addressing early signs and symptoms of psychosis can lead to better outcomes (8). However, few studies have examined directly the relationship between schizotypal traits and sleep problems.

Research has also found a close correlation between alexithymia and sleep problems (9). Alexithymia is a type of emotional disorder, and the alexithymia personality construct includes the following features: difficulty in identifying and describing feelings, difficulty in distinguishing between feelings and bodily sensations, a constricted imagination, and an externally oriented cognitive style (10). Researchers have recently suggested that internalized psychic conflicts and an inability to verbalize these problems result in increased nocturnal arousal and subsequent insomnia in individuals with alexithymia (11). Moreover, a recent study has shown that difficulties in identifying feelings are related to increased general sleep experiences such as hallucinations, frequent waking, and insomnia, leading to the suggestion that general sleep experiences represent a nocturnal manifestation of unprocessed emotions (12); the same study also found that externally oriented thinking is related to decreased general sleep experiences. Meanwhile, schizophrenia also has a strong correlation with alexithymia (13). A considerable amount of research has found that schizophrenia patients exhibit higher levels of alexithymia, and that individuals with higher levels of alexithymia may be more prone to schizophrenia spectrum disorders (14).

Although there has been much research on the relationship between alexithymia and sleep problems, as well as on the relationship between schizophrenia and alexithymia, a review of the literature shows that there have been few studies on the relationships between schizophrenia, alexithymia, and sleep problems. The role of alexithymia in the relationship between schizophrenia and sleep problems is therefore unclear. The purpose of the current investigation was to explore the relationships between schizotypal traits, alexithymia, and sleep problems among college students. We hypothesized that schizotypal traits play an important role in the sleep problems of college students, and that alexithymia is a mediating variable between the two.

## Materials and Methods

### Participants

In this study, 2,811 questionnaires were issued to first-year students at a medical university in Guangzhou, Guangdong, China, of which 2,626 were returned as valid and analyzed, showing an effective response rate of 93.42%. Of the 2,626 participants, 1,601 were female and 1,025 male, with a mean age of 18.34 years (*SD* = 0.83). The study was approved by the ethics committee of Southern Medical University and all participants provided informed consent.

### Measures

#### Schizotypal Personality Questionnaire (SPQ)

The SPQ is a 74-item self-report questionnaire consisting of three dimensions: positive, negative, and disorganized (15). Participants respond to statements with “yes” or “no” answers, depending on their agreement with each item. A “yes” is scored as 1, a “no” is scored as 0, and the total score ranges from 0 to 74. The higher the score, the higher the levels of schizotypal personality traits. According to Raine (16), people scoring in the top 10% can be identified as exhibiting schizotypal traits. A threshold of 36 is considered an acceptable standard in China and has been used in our previous studies [e.g., (17, 18)]. In our sample, 91 individuals who scored higher than 36 were classified as individuals with schizotypy. The Chinese version was revised by researchers in Taiwan and has good reliability and validity, with Cronbach's alpha ranging from 0.9 to 0.91 (19).

#### Toronto Alexithymia Scale (TAS-20)

The TAS-20 consists of 20 items and measures three dimensions of the alexithymia construct: difficulty identifying feelings, difficulty describing feelings, and externally oriented thinking (20). Participants respond to items on a five-point scale, ranging from 1 (“strongly disagree”) to 5 (“strongly agree”). The total score ranges from 20 to 100. For the English version of the scale, cutoff scores have been established empirically; total scores >60 indicate a high degree of alexithymia and scores <52 indicate a definite absence of alexithymia (21). However, the cutoff score must be determined as appropriate in the Chinese population and we referred to the criteria from the Chinese translation of the 20-item TAS. For the TAS-20-C, it is preferable for researchers to use alexithymia as a dimensional variable rather than to apply categorical cutoff scores (22). The Cronbach's alpha for this scale in a student sample is 0.79, indicating good reliability and validity (22).

#### Insomnia Severity Index (ISI)

The ISI consists of seven items and was developed by Bastien et al. (23). It has been used widely to assess the severity of insomnia symptoms. Participants respond to items on a five-point scale, scored on a range of 0–4 (0 = “not at all”; 1 = “mild”; 2 = “moderate”; 3 = “severe”; and 4 = “extremely severe”) (24). The total score ranges from 0 to 28. A score of 0–7 represents no clinically significant insomnia; a score of 8–14 indicates mild insomnia, a score of 15–21 indicates moderately clinical insomnia, and a score of 22–28 indicates severe clinical insomnia. The Cronbach's alpha of this scale is 0.83, indicating high internal consistency (23).

### Data Analysis

Statistical analyses were performed using SPSS 23.0. First, a Harman single-factor test was used on all the variables combined in the questionnaire for factor analysis. The results showed that 15.03% of the variation was explained by the first principal component, which is lower than the critical value (40%) and indicates that there is no common method bias effect among the variables measured in this study. Second, associations between variables were tested with Pearson correlations. AMOS 20.0 was then used to fit the mediation model. The bootstrap mediation technique was adopted to conduct 5,000 samples with replacement, and the maximum likelihood estimation method was used to fit the global model; *p* < 0.05 was used to confirm statistical significance.

## Results

### Prevalence of Schizotypal Traits, Alexithymia, and Insomnia

As shown in Table 1, 91 college students with high schizotypal traits were found, accounting for 3.5% of the overall sample. There were 1,466 individuals with low levels of schizotypal traits, accounting for 55.8% of the overall sample. Meanwhile, the numbers of individuals with mild, moderate, and severe insomnia were 1,965, 220, and 14, respectively, accounting for 74.8, 8.4, and 0.5%, respectively, indicating that a relatively large percentage of the participants had mild insomnia.

**Table 1:** Sample screening status.

	**Groupings**	**Number of students**	**Percentage**
SPQ score	SPQ <14.94 (low schizotypal)	1,466	55.8%
	SPQ ≥ 36 (high schizotypal)	91	3.50%
ISI score	ISI ≤ 7 (no clinical significance)	427	16.30%
	8 ≤ ISI ≤ 14 (mild insomnia)	1,965	74.80%
	15 ≤ ISI ≤ 21 (moderate insomnia)	220	8.40%
	22 ≤ ISI (severe insomnia)	14	0.50%

*SPQ, schizotypal personality questionnaire; ISI, insomnia severity index*.

### Relationships Between Schizotypal Traits, Alexithymia, and Insomnia

The mean scores for the SPQ, TAS-20, and ISI were 14.94 (*SD* = 9.38), 54.77 (*SD* = 6.82), and 10.41 (*SD* = 2.98), respectively. Pearson correlations were used to explore the relationships between schizotypal traits, alexithymia, and insomnia, as shown in Table 2. As predicted, the total SPQ score was significantly positively correlated with the ISI score, the TAS-20 score, and the scores for each dimension of the alexithymia construct (*p* < 0.01). The ISI score was also significantly positively correlated with the TAS-20 score and the scores for each alexithymia dimension (*p* < 0.01).

**Table 2:** Descriptive statistics and correlation analysis for SPQ, TAS-20, and ISI.

**Variable**	**M ± SD**	**SPQ total**	**SPQ pos**	**SPQ neg**	**SPQ dis**	**TAS-20 total**	**DIF**	**DDF**	**EOT**	**ISI total**
SPQ Total	14.94 ± 9.38	1								
SPQ Positive	6.92 ± 4.15	0.812^**^	1							
SPQ Negative	5.55 ± 5.02	0.868^**^	0.511^**^	1						
SPQ Disorganized	3.24 ± 2.93	0.835^**^	0.564^**^	0.624^**^	1					
TAS-20 Total	54.77 ± 6.82	0.581^**^	0.427^**^	0.540^**^	0.493^**^	1				
DIF	14.92 ± 4.50	0.606^**^	0.444^**^	0.555^**^	0.523^**^	0.856^**^	1			
DDF	13.10 ± 2.19	0.455^**^	0.295^**^	0.454^**^	0.393^**^	0.767^**^	0.595^**^	1		
EOT	26.74 ± 2.80	0.084^**^	0.096^**^	0.069^**^	0.050^**^	0.459^**^	0.011	0.128^**^	1	
ISI Total	10.41 ± 2.98	0.454^**^	0.312^**^	0.430^**^	0.404^**^	0.410^**^	0.486^**^	0.306^**^	−0.021	1

*SPQ, schizotypal personality questionnaire; Pos, positive; Neg, negative; Dis, disorganized; TAS-20, Toronto Alexithymia Scale; DIF, difficulty feelings; DDF, difficulty describing feelings; EOT, externally oriented thinking; ISI, insomnia severity index*.

** *p < 0.01*.

### The Mediating Effect of Alexithymia

#### The Mediating Effect of Alexithymia on the Total SPQ Scores and ISI Scores

**Figure 1:**
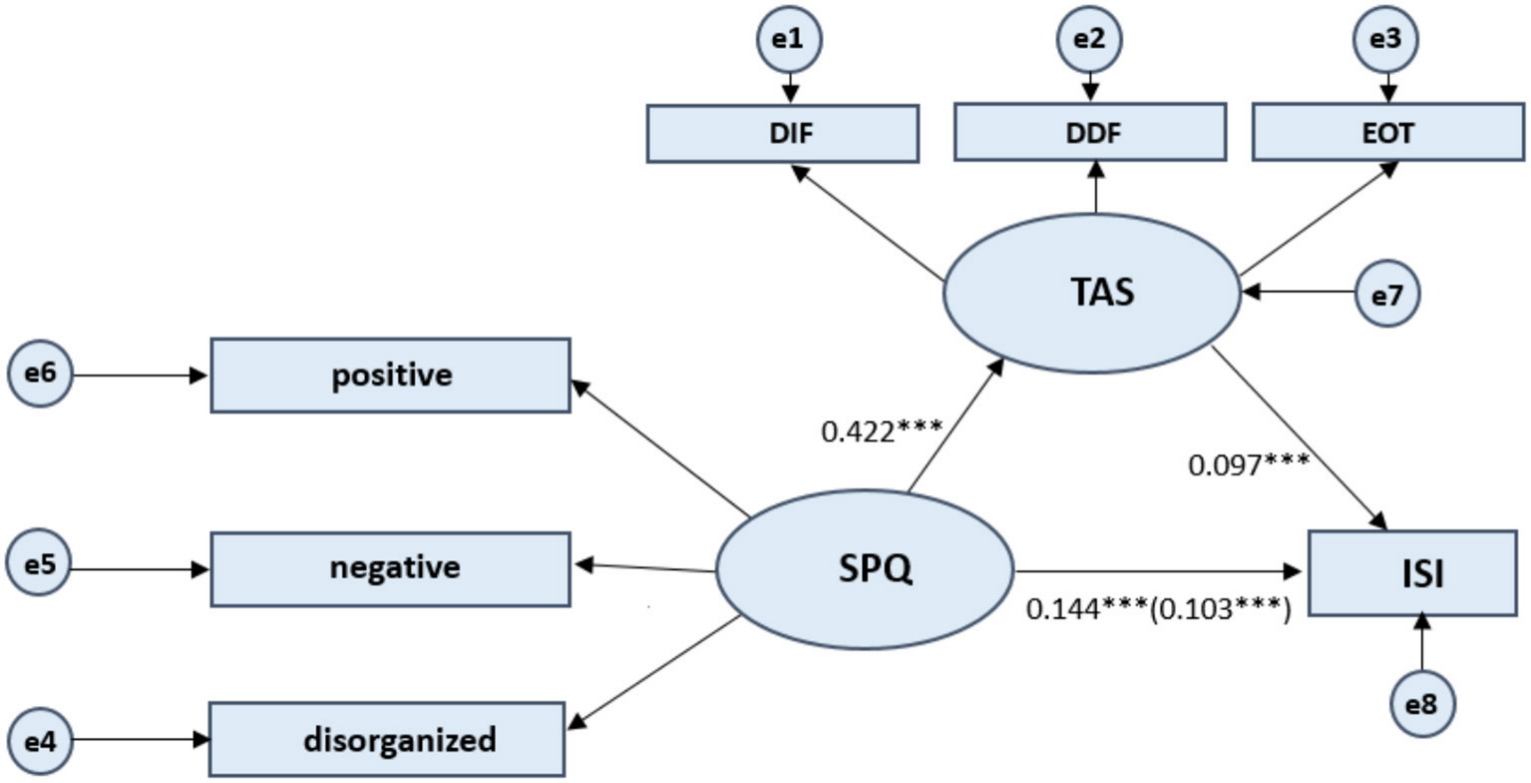
Test of mediation model. Schizotypal Personality Questionnaire (SPQ) consists of three dimensions: positive, negative, and disorganized. Toronto Alexithymia Scale (TAS-20) measures three dimensions of the alexithymia construct: difficulty identifying feelings (DIF), difficulty describing feelings (DDF), and externally oriented thinking (EOT). ISI refers to Insomnia Severity Index. Values presented are standardized regression coefficients. The value in parentheses represents the coefficient for the direct path. e1 to e8 represents each error. Latent Variables use ellipses and Observable Variables use rectangular boxes. ***p < 0.001.

The mediating effect model was constructed by taking the total ISI score as the dependent variable, the total SPQ score as the independent variable, and the total TAS-20 score as the mediating variable (see Figure 1). The process of testing the mediating effect then followed the procedure proposed Wen and Ye (25). In the first step, to conduct regression analysis and test coefficient *c*, the total SPQ score was taken as the independent variable and the total ISI score as the dependent variable. Second, to conduct the regression analysis and test coefficient *a*, the total SPQ score was taken as the independent variable and the total TAS-20 score as the dependent variable. Third, to conduct the regression analysis and test coefficient *b*, the total SPQ score and total TAS-20 score were taken as the independent variables, and the total ISI score was taken as the dependent variable. As shown in Table 3, the results revealed that the coefficients *a, b*, and *c* were all significant, indicating that alexithymia is a mediating variable between schizotypal traits and insomnia; i.e., schizotypal traits were found to affect sleep problems through alexithymia.

**Table 3:** Test of mediation effect of alexithymia on relationship between schizotypal trait and insomnia.

**Procedure**	**Dependent variable**	**Independent variable**		***B***	***SE***	***t***	***R***	***R^**2**^***	***F***
Step 1	ISI	schizotypal trait	c	0.144	0.006	26.09^***^	0.454	0.206	681.113^***^
Step 2	TAS-20	schizotypal trait	a	0.422	0.0115	36.608^***^	0.581	0.338	1340.132^***^
Step 3	ISI	alexithymia	b	0.097	0.009	10.569^***^	0.488	0.238	410.781^***^
		schizotypal trait	c′	0.103	0.007	15.531^***^			

*TAS-20, Toronto Alexithymia Scale; ISI, insomnia severity index*.

*** *p < 0.001*.

Due to these regressions being significant, the indirect effect of alexithymia was tested using the bias-corrected bootstrap method. As shown in Table 4, the results show, first, that the bootstrap 95% confidence interval for the total effect of the model did not contain a zero value [B = 0.144, 95% CI = (0.133, 0.155)]. Second, the direct effect of schizotypal traits on the insomnia severity index did not include a zero value [B = 0.103, 95% CI = (0.090, 0.116)]; and, third, the bootstrap 95% confidence interval for indirect effect did not contain a zero value [B = 0.041, 95% CI = (0.032, 0.049)]. This further indicates that alexithymia had a significant mediating effect on the relationship between schizotypal traits and sleep problems. The effect size of the mediating effect was 28.47%.

**Table 4:** Bias-corrected bootstrap test of indirect effect of alexithymia on relationship between schizotypal trait and insomnia.

	**Effect of value**	**Boot standard error**	**Boot CI limit**	**Boot CI ceiling**
Total effect	0.144	0.005	0.133	0.155
Direct effect	0.103	0.007	0.090	0.116
Indirect effect	0.041	0.004	0.032	0.049

#### The Mediating Effect of Alexithymia on Each Dimension of the SPQ Scores and ISI Scores

**Figure 2:**
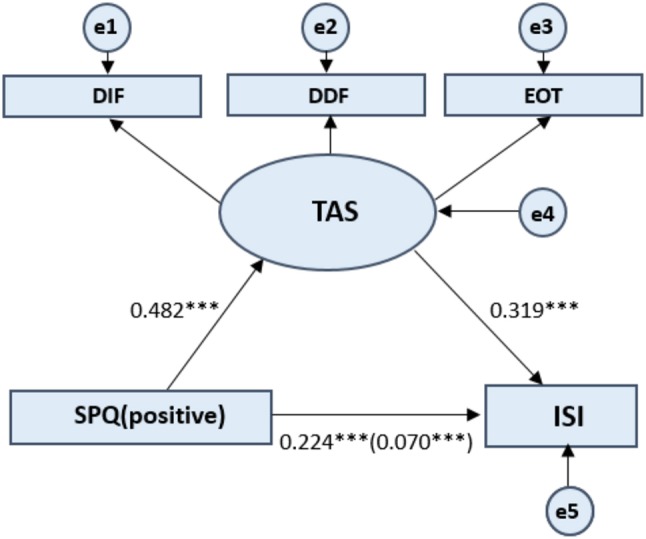
Standardized beta coefficient in TAS partially mediated pathway from SPQ (positive) to ISI. SPQ (positive) represents the positive dimension of the Schizotypal Personality Questionnaire. Toronto Alexithymia Scale (TAS-20) measures three dimensions of the alexithymia construct: difficulty identifying feelings, difficulty describing feelings, and externally oriented thinking. ISI refers to Insomnia Severity Index. The value in parentheses represents the coefficient for the direct path. e1 to e5 represents each error. Latent Variables use ellipses and Observable Variables use rectangular boxes. ***p < 0.001.

**Figure 3:**
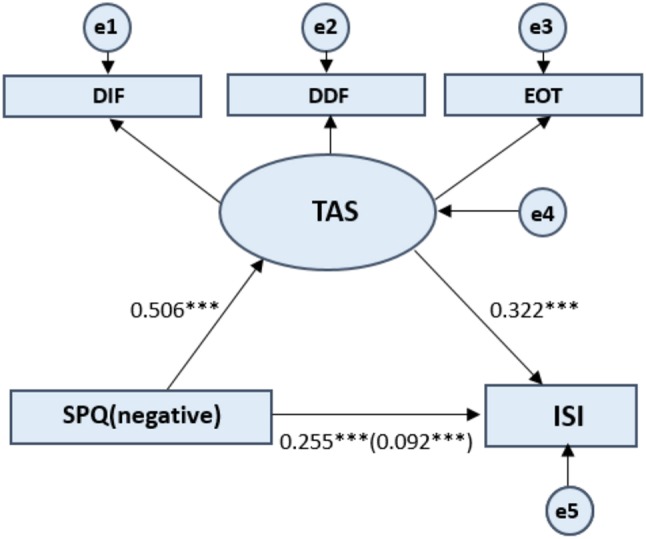
Standardized beta coefficient in TAS partially mediated pathway from SPQ (negative) to ISI. SPQ (negative) represents the negative dimension of the Schizotypal Personality Questionnaire. Toronto Alexithymia Scale (TAS-20) measures three dimensions of the alexithymia construct: difficulty identifying feelings, difficulty describing feelings, and externally oriented thinking. ISI refers to Insomnia Severity Index. The value in parentheses represents the coefficient for the direct path. e1 to e5 represents each error. Latent Variables use ellipses and Observable Variables use rectangular boxes. ***p < 0.001.

**Figure 4:**
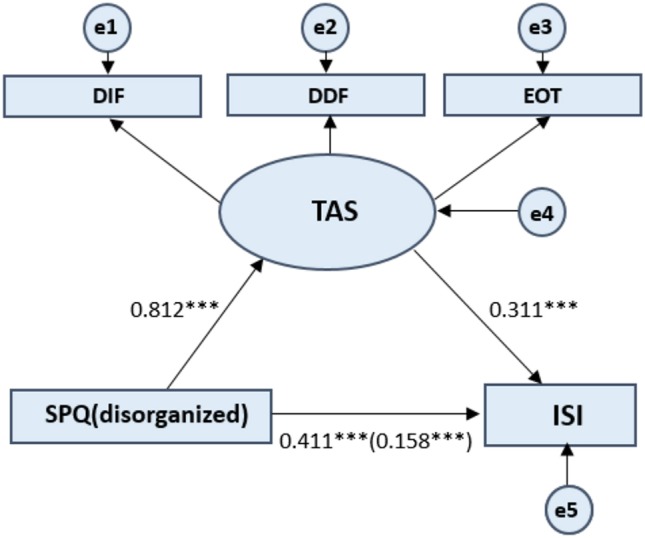
Standardized beta coefficient in TAS partially mediated pathway from SPQ (disorganized) to ISI. SPQ (disorganized) represents the disorganized dimension of the Schizotypal Personality Questionnaire. Toronto Alexithymia Scale (TAS-20) measures three dimensions of the alexithymia construct: difficulty identifying feelings, difficulty describing feelings, and externally oriented thinking. ISI refers to Insomnia Severity Index. The value in parentheses represents the coefficient for the direct path. e1 to e5 represents each error. Latent Variables use ellipses and Observable Variables use rectangular boxes. ***p < 0.001.

For the SPQ subscales scores, the mediating effect model was constructed by taking the total ISI score as the dependent variable, and the positive SPQ score, negative SPQ score, and disorganized SPQ score as the independent variables, and the total TAS-20 score as the mediating variable (see Figures 2–4). The process of testing the mediating effect was the same as above. As shown in Table 5, for the positive dimension, the results revealed that the total effect on the model was 0.224 (*p* < 0.001). The direct effect of the positive dimension of schizotypal traits on the ISI was 0.070 (*p* < 0.001) and the indirect effect was 0.154 (*p* < 0.001). For the negative dimension, the total effect on the model was 0.255 (*p* < 0.001). The direct effect of the positive dimension of schizotypal traits on the ISI was 0.092 (*p* < 0.001) and the indirect effect was 0.163 (*p* < 0.001). For the disorganized dimension, the results revealed that the total effect on the model was 0.411 (*p* < 0.001). The direct effect of the positive dimension of schizotypal traits on the ISI was 0.158 (*p* < 0.001) and the indirect effect was 0.253 (*p* < 0.001). In summary, the results for the three dimensions of the SPQ further confirm the hypothesis that alexithymia has a significant mediating effect on the relationship between schizotypal traits and sleep problems.

**Table 5:** Test of mediation effect of alexithymia on relationship between each dimensions of schizotypal trait and insomnia.

	**Positive dimension**	**Negative dimension**	**Disorganized dimension**
Total effect	0.224	0.255	0.411
Direct effect	0.070	0.092	0.158
Indirect effect	0.154	0.163	0.253

*Schizotypal Personality Questionnaire (SPQ) consists of three dimensions: positive, negative, and disorganized*.

## Discussion

This survey identified that insomnia afflicts 83.7% of a group of first-year students at a medical university, with 74.8% reporting mild insomnia, and 8.9% reporting moderate and severe insomnia. In contrast to a previous study (26), the percentage of first-year students with sleep problems in this study was relatively higher (83.7 vs. 74.00%), which can possibly be explained by the fact that near half of the participants were medical students who with a heavy academic workload in our study. Thus, adaptation to a new environment and the academic workload may have contributed to sleep problems.

The results indicated a positive correlation between schizotypal traits and insomnia severity levels among the students. This is consistent with previous studies (27). Benson (28) found that people with schizophrenia had longer sleep latencies, frequent awakenings, and shorter sleep durations. Another study of schizophrenia patients indicated that most of the participants preferred to stay awake at night and sleep during the day (29). These findings further support the idea that the greater the levels of schizotypal traits, the more severe the sleep problems. Schizophrenia is a complex disorder, caused by both genetic and environmental factors and their interactions (30). It is thought that there is an overlap between the causes of both schizophrenia and sleep problems (31). Compared to people with schizophrenia, sleep problems are relatively easy to study in the general population, and future studies could include the study of the relevant biological factors to elucidate the mechanisms of the link between schizophrenia and sleep problems.

In addition, the results showed a positive correlation between schizotypal traits and alexithymia among the students, which indicated that the greater the levels of schizotypal traits, the more serious the alexithymia. Studies have shown that schizophrenia is a neurocognitive disease, and that abnormalities in key sections of the brain responsible for emotional processing lead to emotional disorders, making it difficult for individuals to process emotions, resulting in alexithymia (32). This explains why individuals with greater levels of schizotypal traits also show more serious alexithymia.

The structural equation modeling further demonstrated that alexithymia played a partial mediating role between schizotypal traits and sleep problems among the students. This means that schizotypal traits could predict directly the sleep quality of the students. However, schizotypal traits also exerted an indirect influence on sleep problems through the mediating effect of alexithymia. This appears to be consistent with some previous studies. For instance, Rehman et al. suggested that sleep problems are associated with psychotic disorders such as paranoia, and that alexithymia may be a potentially important mediating variable (33). However, other studies have shown that, although alexithymia is significantly correlated with sleep symptoms such as prolonged sleep latencies and frequent awakenings, these correlations disappear when the effects of depression are excluded (34). This suggests that sleep problems may also be associated with mood disorders such as depression.

The mediating effect of alexithymia on each dimension of the SPQ and the ISI scores demonstrates that all the three dimensions exerted influences on insomnia through alexithymia, but the disorganized dimension had the greatest impact. This seems to be consistent with some previous studies. For example, negative symptoms are correlated with generalized impairment (35), psychomotor speed, and poor attention (36, 37). This suggests that negative symptoms may be associated with alexithymia (38) and rapid eye movement sleep abnormalities (39). In addition, positive symptoms are correlated with verbal memory and language comprehension deficits (40) which can lead to inner emotions being difficult to release and deal with, resulting in insomnia at night. Finally, disorganized symptoms are correlated with global impairment, as well as language and memory problems (41). This means that disorganized symptoms experiences almost all of these cognitive impairment, including those that have occurred on both the positive and negative dimensions. Therefore, the disorganized dimension has the greatest impact on sleep problems through the partial mediation effect of alexithymia.

In any case, the causes of sleep problems still need further exploration, including investigation of the impact of depression on alexithymia, and the independent effect of other emotional disorders. Additionally, in order to improve the sleep quality of college students, and to enhance the pertinence and effectiveness of psychological health education, findings from the current study may suggest that psychological counseling might help students develop the ability to express their inner feelings and to understand others' emotions from the perspective of alexithymia.

The limitations of this study are as follows. First, this is a cross-sectional study, so it is still not known how is the dynamic relationship among alexithymia, schizotypal traits and sleep. Future study need to address this issue. Second, alexithymia is generally assessed by self-report or interview, but individuals with schizotypal traits often have low levels of self-awareness. Relying on these subjective approaches can, therefore, be problematic. In future research, self-report approaches should be combined with objective measures. Third, the evaluation of sleep quality in our study relied on the single source of a self-report questionnaire. The data obtained could be combined with polysomnography and the use of sleep diaries to obtain more comprehensive sleep data. Lastly, the participants surveyed in the current study were all college students. Therefore, they are not sufficiently representative of the general population, and the extrapolation validity of this research is poor. To be more representative of the general population, future research would need to replicate the results using larger samples, and the samples expanded to include different demographic groups.

In conclusion, we found positive correlations between schizotypal traits, alexithymia, and sleep problems, while alexithymia was found to partially mediate the relationship between schizotypal traits and sleep problems. We may be able to improve the sleep quality of those with a high level of schizotypal traits by training their ability to express emotion.

## Data Availability Statement

The datasets generated for this study are available on request to the corresponding author.

## Ethics Statement

The studies involving human participants were reviewed and approved by the Ethics Committees of Southern Medical University. Written informed consent to participate in this study was provided by the participants' legal guardian/next of kin.

## Author Contributions

QM and XZ collected and analyzed the data, and wrote the first draft of the manuscript. LZ generated the idea, designed the study, interpreted the data, and wrote the manuscript.

### Conflict of Interest

The authors declare that the research was conducted in the absence of any commercial or financial relationships that could be construed as a potential conflict of interest.
